# Extensin-like Protein OsPEX1 Modulates Grain Filling in Rice

**DOI:** 10.3390/plants14172723

**Published:** 2025-09-01

**Authors:** Na Liu, Jieni Li, Cong-Cong Wang, Tingting Yang, Ao Li, Peng Zeng, Haifeng Peng, Yuexiong Zhang, Dahui Huang, Xia Zheng, Xiang-Qian Zhang

**Affiliations:** 1 Department of Horticulture, School of Agricultural and Biological Engineering, Foshan University, Foshan 528000, China; 19112181874@163.com (N.L.); jennylee_st@foxmail.com (J.L.); 18853195727@163.com (A.L.); pengzeng@fosu.edu.cn (P.Z.); 2 Rice Research Institute, Guangxi Academy of Agricultural Sciences/Guangxi Key Laboratory of Rice Genetics and Breeding, Nanning 530007, China; zhangyx1518@126.com (Y.Z.); hdh1103@163.com (D.H.); 3 College of Life Sciences, South China Agricultural University, Guangzhou 510642, China; zhizhiping2356@163.com (C.-C.W.); ytting@hnu.edu.cn (T.Y.); phf72@scau.edu.cn (H.P.); 4 Institute of Bast Fiber Crops, Chinese Academy of Agricultural Sciences, Changsha 410205, China

**Keywords:** rice, extensin, grain filling, endoplasmic reticulum

## Abstract

Grain filling is a vital factor influencing both rice grain yield and quality, yet its underlying mechanisms remain poorly understood. In this study, we perform a functional analysis of the grain-filling defective mutant *pex1* in rice. *pex1* plants produce seeds that are floury, thick-branched, and exhibit a significantly slower grain-filling rate compared to the wild type. Further analysis reveals that the *pex1* mutants accumulated more starch in the pericarp but exhibited a defect in starch accumulation in the endosperm during grain filling, indicating an impaired transport of photosynthetic products from the pericarp to the endosperm. Cells within the nucellar projection in the *pex1* mutant appear irregular and loose loosely arranged, consistent with defective transfer of assimilates. Expression analysis reveals a downregulation of key grain-filling genes during the filling phase in the *pex1* mutant compared to the wild type, which correlates with the reduced grain-filling rate. Subcellular localization suggests that OsPEX1 is associated with the endoplasmic reticulum. Our findings demonstrate that *OsPEX1* plays a crucial role in grain filling.

## 1. Introduction

Grain filling constitutes a pivotal stage in seed development, directly impacting grain weight and rice quality. Following flowering, pollination, and double fertilization, the rice ovary transforms into a caryopsis, initiating the grain-filling phase. During this period, the photoassimilates from leaves are transported to caryopsis, ultimately leading to grain maturation. The rice caryopsis comprises complex maternal and filial tissues [1]. Unlike other agronomic traits of rice, grain filling of rice caryopses exhibits intricate temporal and spatial dynamics alongside environmental variability [1].

Grain fill can be categorized into two major and sequential phases—pre-fill and fill phases [2,3]. Pre-fill is characterized by cell division, expansion, and differentiation that creates the structural framework for subsequent storage product accumulation--the fill phase. Symplasmic unloading appears to operate in both pre-fill and fill phases in rice grains. Sucrose is the major phloem-imported solute contributing to seed development. For pre-fill, upon reaching the caryopsis via the phloem of the dorsal vascular bundle, sucrose undergoes partial hydrolysis by cell wall invertases (cwINVs; specifically, OsGIF1/OsCIN2) into glucose and fructose [2,4]. The cellular distribution of SWEETs (sugars will eventually be exported transporter 11, 14, and 15) and cwINVs in grain maternal tissues are strikingly co-localized. As a result, phloem-imported sucrose, released at specific cellular sites to the grain apoplasm, is hydrolyzed to its hexose moieties [2]. These are retrieved by hexose transporters located in filial (aleurone—SWEET4, monosaccharide transporters MST4 and MST6) tissues [2]. The hexoses function as signals and fuel to drive the development of the pre-fill caryopsis [2]. During grain fill, expression levels of maternal SWEET11 and SWEET15 are strongly elevated (largely in the nucellus) while cwINVs, MST4/6, and SWEET4 expression decline markedly in the aleurone [2]. SWEET4 and MST4/6 are replaced by SUTs (particularly SUT1) that retrieves apoplasmic sucrose and are major contributors to endosperm starch accumulation [2]. Researchers have focused on identifying the components involved in grain fill [5]; however, information regarding the process of grain filling remains limited, particularly in rice.

Grain filling represents a complex trait that encompasses the loading, transfer, and exchange of nutrients between maternal and filial tissues within the developing caryopsis. The structure of the developing caryopsis includes various tissue types: the embryo, diploid filial tissue, and endosperm--a triploid filial tissue comprising an inner starchy endosperm and an outer aleurone layer. Surrounding the endosperm and embryo are the diploid maternal tissues, including the pericarp, seed coat, vascular bundle, and nucellus tissues [6]. In plants, sugars are transported through apoplasmic or symplasmic pathways facilitated by plasmodesmata [7]. Plentiful plasmodesmata are present between adjacent cells in maternal tissues, spanning from the vascular parenchyma to the nucellus within rice caryopses. Assimilates originating from sieve element companion cell complexes must traverse the vascular parenchyma, pigment strand, and nucellus via plasmodesmata before reaching the aleurone layer [8]. Conversely, filial tissues, encompassing both the endosperm and embryo, lack symplastic connections with the maternal tissues in cereal crops [9]. Notably, there are limited plasmodesmata between the nucellus and aleurone layer in rice caryopses [7]. Consequently, the traditional understanding posited assimilates following a symplastic pathway between the phloem and nucellus and then an apoplasmic pathway from the maternal and filial interface [7]. Nevertheless, the precise mechanisms governing symplastic translocation for assimilate transport in rice caryopses remain inadequately elucidated.

Grain size is closely associated with the grain-filling process. Genes that regulate grain size determine the potential storage capacity during this phase [1]. Given the critical importance of grain size as a determinant of sink capacity in crop plants, researchers have focused on identifying the components involved in grain size regulation [5]. *OsPEX1*, a leucine-rich repeat extensin (LRX) gene, affects grain cell expansion, primarily impacting grain width, as well as the biosynthesis of amino acids, amylose, and storage proteins [10]. Notably, *OsPEX1* is highly expressed in the dorsal vascular bundle, peaking at 7 days after pollination (DAP) [10], suggesting its role in regulating post-phloem nutrient flow to developing rice grains. This study investigates the function of *OsPEX1* in grain filling. Our findings demonstrate that the LRX protein OsPEX1 plays a crucial role in grain filling.

## 2. Results

### 2.1. The pex1 Mutant Shows Low Rate of Grain Filling

**Figure 1:**
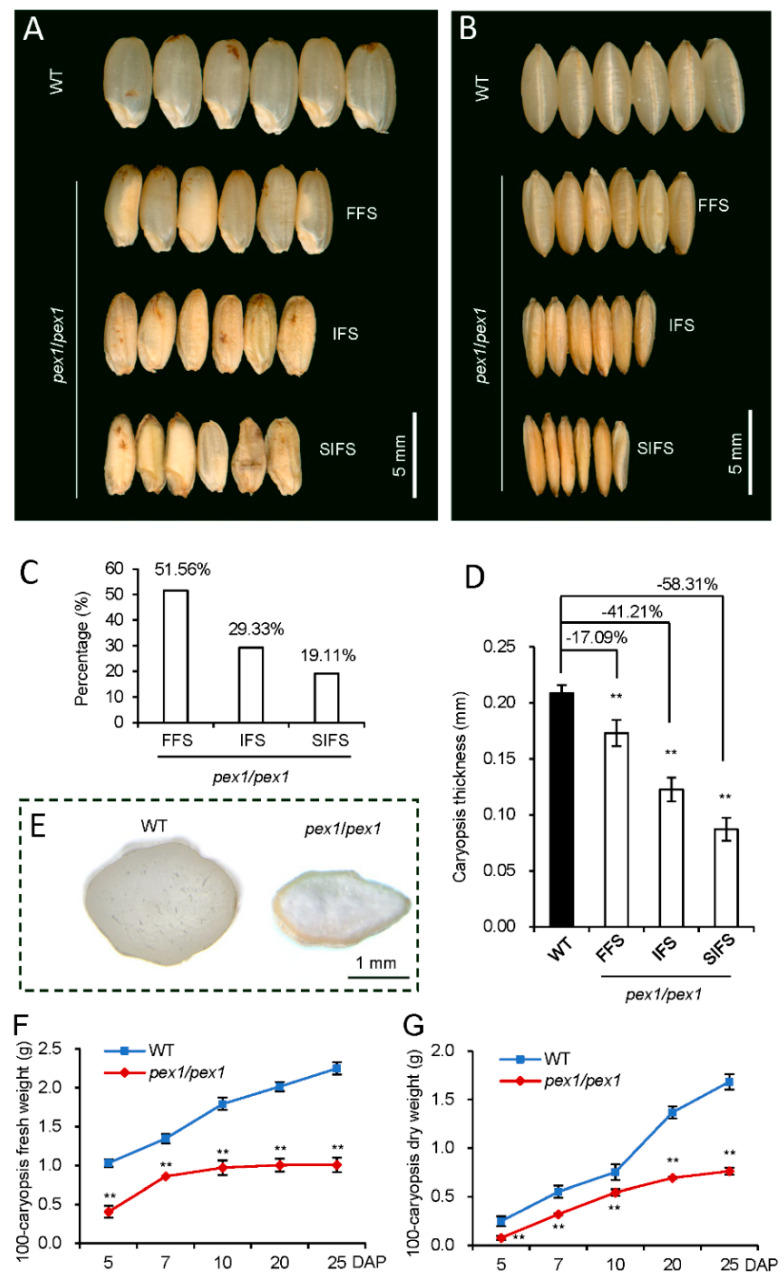
Phenotype analysis of *pex1* mutant seeds. Comparison of WT and *pex1* mature seeds showing various degrees of grain-filling defects on the lateral (**A**) and dorsal (**B**) sides. (**C**) Percentage of different types of caryopses. (**D**) Comparison of caryopsis thickness between *pex1* mutants and WT. The thickness of the caryopsis was measured at its thickest point using a vernier caliper. (**E**) Transverse sections of fully filled seeds from WT and *pex1* mutant. FFS, fully filled seeds; IFS, incompletely filled seeds; SIFS, severely incompletely filled seeds. (**F**,**G**) Trends in grain fresh weight (**F**) and dry weight (**G**) of WT and *pex1* mutant during grain filling. All values are means ± SD (n = 100). **, *p* < 0.01, determined by Student’s *t*-test. DAP, days after pollination.

We previously identified a Ds insertion mutant characterized by small mature caryopses [10]. This Ds insertion represented an activating mutation, leading to elevated *OsPEX1* levels in the mutant compared to the wild type (WT) [10,11]. Surprisingly, upon examining the seeds of the mutant plants, we noted that 49.44% of *pex1* seeds exhibited incomplete filling and a shrunken surface, while nearly all WT seeds were completely filled (Figure 1A–C). The impaired grain filling in *pex1* plants resulted in reduced caryopses thickness compared to the WT (Figure 1D). Further scrutiny revealed that approximately 52% of the “fully filled” *pex1* seeds also displayed defective filling phenotypes, such as floury endosperm (Figure 1E).

We next investigated the filling dynamics of the *pex1* caryopsis. At the initial stage of caryopsis development, both *pex1* and WT demonstrated a rapid increase in caryopsis weight (Figure 1F,G). While there was reduced biomass at 5–10 days after pollination (DAP), the rate of biomass accumulation by the mutant is identical to that of the WT. In contrast to pre-fill, biomass accumulation rates are substantially different between the mutant and WT at the fill phase. The caryopsis weight in the *pex1* mutant exhibited slow growth from 10 DAP, indicating inhibited endosperm formation; conversely, the WT displayed a steady increase over the 25-day filling period (Figure 1F,G). These results highlight the significantly slower grain filling rate in the *pex1* compared to the WT during grain filling.

### 2.2. The pex1 Mutant Displayed Abnormal Starch Accumulation in the Pericarp

**Figure 2:**
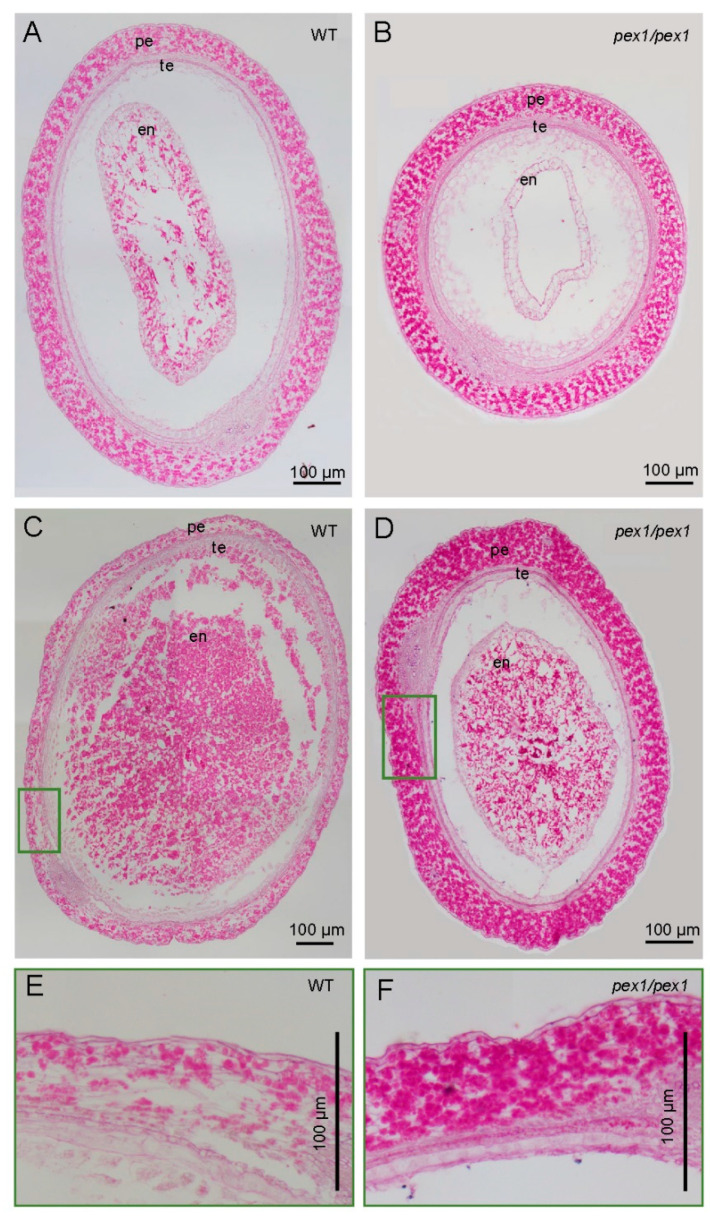
Substantial starch accumulation in the pericarp of *pex1* mutant. Transverse sections of WT (**A**) and *pex1* (**B**) caryopsis at 5 DAP stained with PAS. (**C**,**D**) Transverse sections of WT (**C**) and *pex1* (**D**) caryopsis at 7 DAP stained with PAS. (**E**,**F**) Magnified views of the green boxes area in (**C**,**D**). Pink color corresponds to PAS-stained starch. pe, pericarp (fruit coat); te, testa (seed coat); en, endosperm.

To explore the physiological functions of *OsPEX1* in grain filling, we analyzed starch accumulation in the pericarps of both WT and *pex1* at 5 DAP and 7 DAP. Microscopic examination of grain sections stained with periodic acid–Schiff (PAS) revealed minimal starch content in the pericarp of WT (Figure 2A,C,E). Conversely, substantial starch accumulation was observed in the pericarp of *pex1* (Figure 2B,D,F). Also, the *pex1* mutant exhibited reduced starch content in the endosperm compared to WT. These findings indicate a possible role of *OsPEX1* in regulating sugar transport in developing rice grains. Additionally, the entire pericarp of WT tended to become thinner at 7 DAP compared to 5 DAP, whereas the corresponding region of *pex1* showed no discernible difference between 5 DAP and 7 DAP (Figure 2A–D).

**Figure 3:**
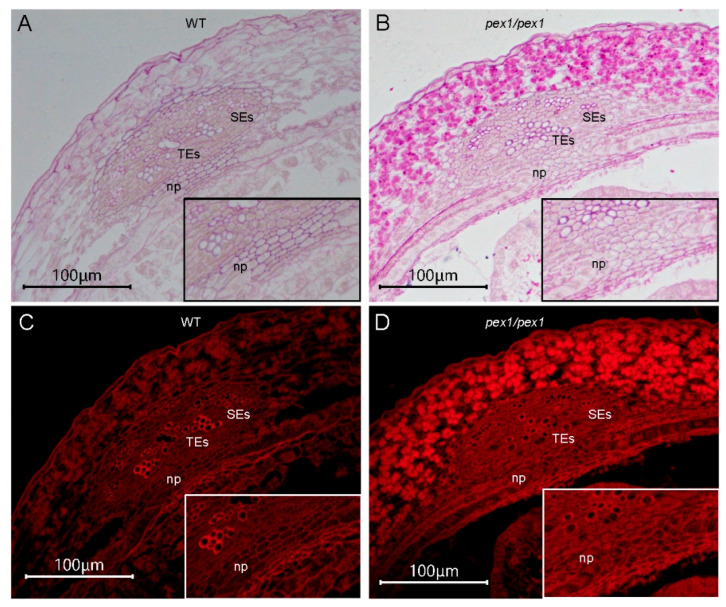
Morphological characteristics of dorsal vascular bundle and nucellar projection from WT and *pex1* at 7 DAP after PAS staining. The maternal tissue at the dorsal positions with a vascular bundle of WT and *pex1* caryopsis are observed under white light (**A**,**B**) and red fluorescence (**C**,**D**). Insets in (**A**–**D**) correspond to the nucellar projection that is magnified 1.4 times. TEs, tracheary elements; SEs, sieve elements; np, nucellar projection. Red fluorescence was detected using a filter set with 560 nm excitation and 635 nm emission.

The dorsal vascular bundle serves as the primary pathway for sugar delivery to the developing caryopsis [9]. Additionally, the nucellar projection plays a crucial role in the import of sugar into the developing endosperm. To further explore the functions of *OsPEX1* in grain filling, we examined the morphological characteristics of the dorsal vascular bundle and nucellar projection at 7 DAP using PAS staining. The cell walls of tracheary elements (TEs) and sieve elements (SEs) in the dorsal vascular bundle of the *pex1* mutant appeared to thicken, unlike those in the WT (Figure 3A,C). Cells within the nucellar projection were orderly and compactly arranged in the WT, whereas they exhibited disarray and lack of organization in the *pex1* mutant (Figure 3B,D), which might block sugar and water from *pex1* maternal tissue to the endosperm.

To gain a more detailed insight into the expression localization of *OsPEX1*, GUS staining for *OsPEX1* promoter activity in ZH11 was observed. Promoter-GUS fusion analysis revealed that *OsPEX1* was expressed in nucellar epidermis and projection cells (Figure S1).

### 2.3. The pex1 Exhibits Thickened Bran at Mature Caryopsis

**Figure 4:**
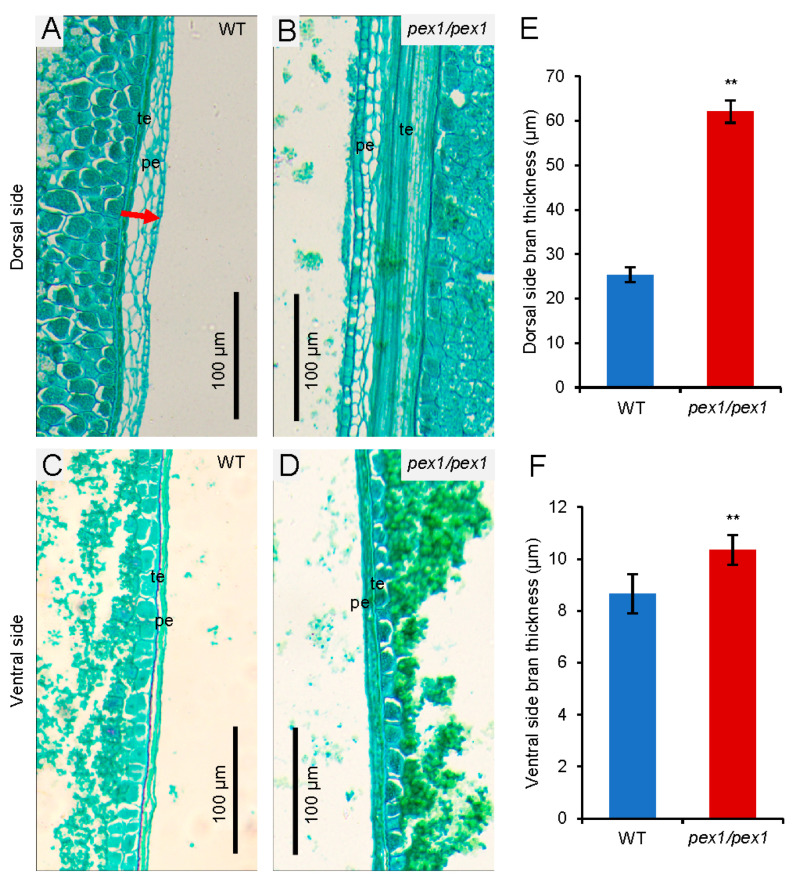
*OsPEX1* plays an important role in caryopsis bran development during grain filling. Longitudinal section of mature caryopsis on the dorsal side (**A**,**B**) and ventral side (**C**,**D**) stained with safranine O/fast green. (**E**) The thickness of dorsal side bran (pericarp, testa). (**F**) The thickness of ventral side bran. Bran thickness means pericarp thickness plus testa thickness. The red arrow indicates the position of bran thickness. pe, pericarp (fruit coat); te, testa (seed coat). All values are means ± SD (n = 100). **, *p* < 0.01, determined by Student’s *t*-test.

The *pex1* mutant displays thickened pericarps in the early stage of caryopsis development compared to the WT (Figure 2A–D). To delve deeper into the involvement of *OsPEX1* in pericarp development, we analyzed the morphological characteristics of the *pex1* and WT pericarps at the mature stage. As anticipated, the *pex1* mutant exhibited pericarp thickening, particularly at the dorsal side. Notably, the bran thickness of the *pex1* mutant was significantly greater at the dorsal testa compared to that of the wild type (Figure 4A,B,E). Likewise, the ventral pericarp and testa of the *pex1* mutant displayed significantly greater thickness compared to those of the wild type (Figure 4C,D,F).

### 2.4. OsPEX1 Is Associated with Endoplasmic Reticulum

**Figure 5:**
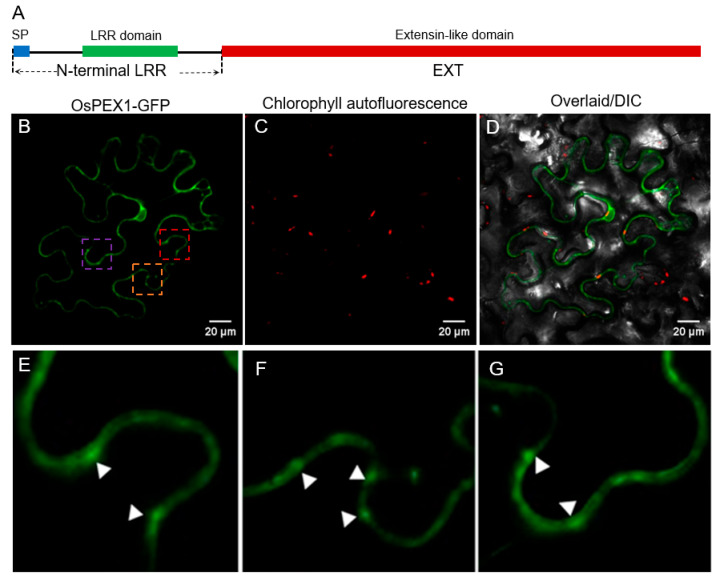
Subcellular localization of OsPEX1 in *N. benthamiana* leaf epidermis. (**A**) Schematic representation of the OsPEX1 proteins. SP, signal peptide. LRR, leucine-rich domain. EXT, extension-like protein domain. (**B**–**D**) Cell wall localization of OsPEX1 in *N. benthamiana* leaf epidermis. (**E**–**G**) Magnified views of the red (**E**), orange (**F**), and purple (**G**) box in (**B**). GFP fusions to OsPEX1 proteins are shown in green; plastid autofluorescence is in red. The white triangle indicates that the green fluorescence is distributed in spotty patterns on the cell wall.

Plant LRXs are cell wall-localized chimeric extensin proteins [12]. A recent study showed that AtLRX11 (AtPEX4), an ortholog to OsPEX1, is localized to the cell wall, while its LRR domain is associated with the plasma membrane [13]. However, the manner in which a cell wall protein physically connects to the plasma membrane is questionable. The hypothetical OsPEX1 protein sequence includes a C-terminal extensin-like domain and a distinct N-terminal domain containing leucine-rich repeats (LRRs) and a putative signal peptide at the N-terminus (Figure 5A). We investigate the subcellular localization of OsPEX1. As expected, we found OsPEX1-GFP is localized to the cell wall region (Figure 5B–D). However, the green fluorescence from OsPEX1-GFP was not continuously distributed in the cell periphery but rather spotty (Figure 5E–G). To determine if the LRR of OsPEX1 is also associated with the plasma membrane similar to the AtLRX11, the coding sequence of the fluorescence protein was fused to the C-terminal end of the LRR region containing the signal peptide to generate the LRR-GFP fusion reporter. Co-expression analysis of LRR-GFP with either endoplasmic reticulum marker (ER-mCherry) or plasma membrane marker (PM-mCherry) in rice protoplast confirmed that LRR-GFP was localized to ER rather than PM (Figure S2). Collectively, these results established that LRX protein OsPEX1 is physically associated with ER membranes.

### 2.5. Expressions of Key Genes Related to Grain Filling Were Altered in pex1

**Figure 6:**
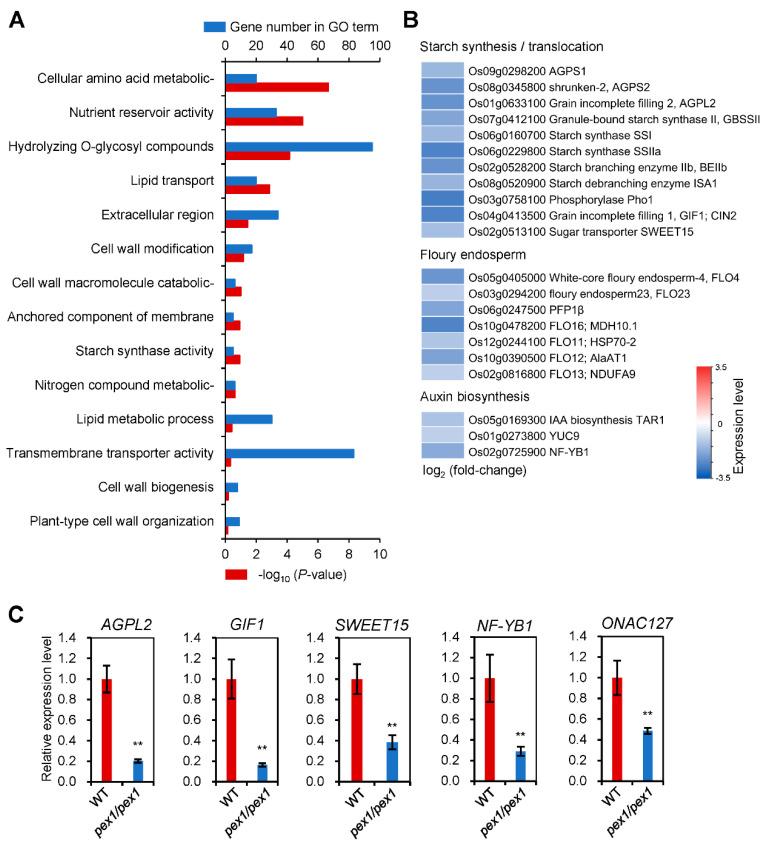
Gene Ontology analysis of the DEGs between wild type (WT) and *pex1* mutant caryopsis at 7 d after pollination (DAP) based on RNA-seq data. (**A**) The significantly enriched GO terms in the DEGs of 7 DAP caryopsis and their associated *p*-values are shown. Lower *x*-axis, −log_10_ (*p*-value); upper *x*-axis, the number of genes with a given GO term. (**B**) Expression of starch synthesis and translocation, floury endosperm, and auxin biosynthesis associated genes quantified by RNA-seq analysis. The log_2_ fold-change values between *pex1* mutant and WT were calculated from RNA-seq data and are shown as a heat map. (**C**) qRT-PCR analysis of grain-filling-related genes. Each column represents the means of three biological samples ± SD. Asterisks represent significant difference determined by Student’s *t*-test at *p*-values < 0.01 (**).

To further investigate the role of *OsPEX1*, we performed RNA sequencing (RNA-Seq) analysis of developing caryopses at 7 d after pollination (DAP) from both WT and *pex1* plants. A total of 6908 differentially expressed genes (DEGs) were identified, including 3458 upregulated and 3450 downregulated genes. The Gene Ontology (GO) study of DEGs at 7 DAP caryopsis unveiled enrichment in terms associated with the cell wall, encompassing ‘cell wall modification’, ‘cell wall macromolecule catabolic process’, ‘cell wall biogenesis’, and ‘plant-type cell wall organization’. Additionally, there was enrichment in GO terms linked to nutrition metabolism and transport like ‘cellular amino acid metabolic process’, ‘nutrient reservoir activity’, ‘lipid transport’, ‘lipid metabolic process’, and ‘starch synthesis activity’ (Figure 6A).

We investigated DEGs associated with starch synthesis and metabolism and found its enzyme-encoding genes, including *ADP-glucose pyrophosphorylase small subunit* (*AGPS*), *ADP-glucose pyrophosphorylase large subunit* (*AGPL2*, also known as *GIF2*), *granule-bound starch synthase II* (*GBSSII*), starch synthases (SS) isoforms, including *SSI* and *SSIIa*, *starch branching enzyme* (*BEIIb*), *debranching enzyme* (*ISA1*), and *starch/a-glucan phosphorylase* (*PHO*) [1], were downregulated in the *pex1* mutant compared to the WT. Additionally, genes related to sugar transport, such as *grain incomplete filling 1* (*GIF1*) and *Sugars Will Eventually be exported Transporter* (*SWEET15*) [1,14], were significantly downregulated in *pex1* caryopses (Figure 6B).

Several genes associated with floury endosperm [15,16,17,18], serving as carbon metabolism regulators, including FLO4, Fructose-6-phosphate-2-kinase/Fructose-2,6-bisphosphatase (F2KP; FLO23), Pyrophosphate: fructose-6-phosphate 1-phosphotransferase (PFP1β), and Alanine Aminotransferase 1 (AlaAT1; FLO12), were markedly downregulated in the pex1 mutant, which aligns with the characteristics of the floury endosperm in pex1 caryopsis (Figure 1F). Additionally, genes involved in the regulation of starch synthesis in rice endosperm, such as FLO11 encoding a heat shock protein 70 (HSP70-2) [19], FLO13 encoding a mitochondrial complex I subunit (NDUFA9) [20], and FLO16 encoding a NAD-dependent cytosolic malate dehydrogenase [21], were also suppressed in pex1 (Figure 6B).

Three primary mechanisms have been proposed to explain the link between auxin and starch [22]. The first mechanism is that auxin upregulates the expression of genes encoding enzymes responsible for catalyzing the conversion of sucrose into starch. Additionally, auxin is essential for transporting sucrose from source leaves to developing grains and is believed to facilitate sucrose transport from source to sink. Auxin plays a positive role in regulating grain filling and the accumulation of storage products. In our study, we observed a downregulation of auxin biosynthesis-related genes, including *tryptophan aminotransferase TAR1*, endosperm-preferential *YUCCAs* like *YUC9*, and *nuclear transcription factor Y subunit B* (*NF-YB1*), in the *pex1* mutant (Figure 6B). This finding aligns with the increase in chalkiness (Figure 1). Our analysis revealed the downregulation of genes associated with cell wall extensibility during seed development in *pex1*, including *EXPB3*, *EXP4*, *EXPA10*, and a pericarp-preferential *NAC* transcription factor *ONAC127* (Figure S3). Furthermore, the expression levels of key genes involved in grain filling were substantially inhibited in *pex1* caryopses, as verified by Figure 6C, consistent with the RNA-Seq analysis results (Figure 6 and Figure S3).

## 3. Discussion

In this study, we characterized the *OsPEX1* gene, one of the eight *LRX* genes in the rice genome [10]. Overexpression of *OsPEX1* resulted in altered plant development from the vegetative stage onward [10,23], affecting seed development and grain filling. The diverse malformations observed in the *pex1* mutant suggest that *OsPEX1* plays essential and fundamental roles in the development of rice plants.

Higher plant *LRX* genes can be divided into two categories based on expression patterns: those predominantly expressed in vegetative tissues and those primarily active in reproductive tissues. These classifications align closely with their phylogenetic clades [12,24]. Interestingly, *OsPEX1*—a member of the pollen-expressed *PEX* subfamily—shows broad expression, with high levels in roots, stems, and developing caryopses but significantly lower levels in leaves and glumes. This expression pattern correlates with the pleiotropic phenotypes of *pex1* mutants, which exhibit dwarfism and small caryopses but minimal effects on leaf and glume development [10,11]. Despite being a direct homolog of *AtLRX11*, *OsPEX1* appears to have functionally diverged, highlighting potential differences in gene regulation between rice and *Arabidopsis*.

There are three different types of grain-filling phenotypes in *pex1* mutant (Figure 1). The different phenotypes of grain filling observed in the homozygous mutant arise from the random development of grains on the primary and secondary branches of the panicle. Generally, grain development on the secondary branches is more sensitive to variations in biomass supply [25]. Additionally, there exists a temporal asynchrony in the development of floral organs. In rice panicles, a time difference of 2–5 days exists between the caryopsis at the top, which opens first, and the caryopsis at the base, which opens later. This timing discrepancy results in varying initiation times for grain filling. Flowers that open first gain priority access to photosynthetic products, while basal grains face disadvantages due to competition for resources [26,27].

### 3.1. OsPEX1 Contributes to Grain Filling

The seed development process in rice comprises three phases closely associated with grain filling. Initially, the ovary rapidly enlarges, reaching the limit of the husk at 6 days after pollination (DAP) [6]. Concurrently, starch granules begin to accumulate in the endosperm. The second phase, known as the linear phase of grain filling, is characterized by rapid accumulation of starch and storage proteins, resulting in an increase in grain width and thickness. Finally, over the course of 30 days, caryopsis development culminates in the formation of a mature grain with maximum weight. The *pex1* mutant exhibited a defect in grain filling during the grain-filling stage (Figure 2), while its pericarp accumulated more starch compared to the equivalent tissue in the WT grains (Figure 3). Given the normal leaf morphology of *pex1* [11], it is hypothesized that the impaired grain filling may result from compromised transport capabilities of assimilated product from the pericarp to the endosperm.

The dorsal vascular bundle plays a crucial role in transporting photosynthetic assimilates to the developing caryopsis [9]. Adjacent to the dorsal vascular bundle, the nucellar projection (NP) is also critical in grain filling [6]. *OsPEX1* exhibits high expression levels in the cells of the vascular bundle [10]. The *pex1* mutant displays abnormal nucellar projection cells (Figure 4), further supporting the notion of hindered transport of assimilates in the *pex1* mutant. The transcriptional levels of starch synthesis-related genes, including *AGPS1*, *AGPS2*, *AGPL2*, *GBSSII*, *SSI*, *SSIIa*, *BEIIb*, and *ISA1*, were lower in *pex1* grains compared to those in WT (Figure 6), which aligns with the reduced filling rate in *pex1*. Additionally, the expressions of the *GIF1* and *SWEET15* genes, which are directly involved in sugar transport, were significantly downregulated in the *pex1* caryopses. In summary, the upregulation of *OsPEX1* disrupts the transport of photosynthetic assimilation products, leading to altered carbohydrate storage in cereals; consequently, more starch is sequestered within the pericarp, limiting its availability for starch synthesis in the endosperm.

### 3.2. Where Is the Subcellular Localization of LRX Proteins

Apart from the intricate membrane signaling system, plants possess cell wall signal transduction pathways, allowing them to sense and respond to alterations in the cell wall state and components, facilitating coordinated plant growth, development, and enhanced adaptability to environmental changes. Increasing evidence suggests that any perturbation in the plant cell wall directly impacts cell membrane systems, and reciprocally, disruption to intracellular components may also influence the plant cell wall [28,29]. Although extensin stands as the most abundant structural protein in the cell wall, its relatively straightforward structure implies a lack of signal transduction function despite its prevalence as a structural component [30]. Recent studies indicate that extension-like proteins may participate in plant cell signal transduction via the LRX-RALF-CrRLK1L pathway [12,31,32]. Given that LRX proteins contain an extensin domain, LRX family proteins are conventionally assumed to be situated in the cell wall, serving as structural components, which contradicts their role in the signal transduction process within plant cells.

Recent findings in *Arabidopsis thaliana* indicate that AtLRX11, an ortholog to OsPEX1, localizes to the cell wall, while its LRR domain is associated with the plasma membrane [13]. This observation suggests that plant LRX proteins do not conform to a typical structural protein profile in the cell wall. These results raise important questions: How does a cell wall protein physically connect to the plasma membrane, and what function does it serve? Intriguingly, our study reveals that OsPEX1 is associated with the endoplasmic reticulum rather than the plasma membrane (Figure 5B). As shown in Figure S4, endoplasmic reticulum is known to interconnect among cells via the ER desmotubule (DT) within the plasmodesma (PD), thus establishing a continuous ER network [33,34]. Plasmodesmata play a crucial role in connecting the protoplasm of neighboring cells, thereby facilitating both material transport and information exchange [35,36]. Fluorescence microscopy has revealed that typical fusion expression of plasmodesma and fluorescent proteins displays a punctate distribution across the cell wall. Given that OsPEX1 is a cell wall-localized protein with a punctate distribution and is associated with the endoplasmic reticulum, it is plausible that OsPEX1 may interact with desmotubule. Consequently, it is reasonable to propose that OsPEX1 may be a DT-localized protein, providing a clear explanation for its role in signal transduction. However, the precise subcellular localization of OsPEX1 remains to be determined.

### 3.3. OsPEX1 Is Involved in Assimilate Transport

The high expression of *OsPEX1* in the ovular vascular trace [10], combined with the reduced filling rate and changes in cellular morphology within the nucellar projection, suggests that *OsPEX1* is involved in the assimilate translocation in caryopses.

Sucrose from rice leaves can be transported into the caryopsis either through symplastic transport facilitated by plasmodesmata or via apoplastic translocation mediated by transporters [7,8]. Once unloaded into the caryopsis during early grain-filling stages, sucrose can be enzymatically hydrolyzed into monosaccharides by GIF1 [4]. This phase coincides with the rapid expansion of the rice ovary immediately after fertilization [37], accompanied by the accumulation of numerous starch granules in the pericarp [37,38], which correlates with caryopsis development and endosperm starch accumulation [39]. During grain filling, assimilates, including sucrose, may traverse from the phloem to parenchyma cells within the ovular vascular bundle, proceeding to the nucellar projection and nucellar epidermis through symplasmic pathways via plasmodesmata. It is plausible that OsPEX1 may be localized in the desmotubules of the plasmodesma, thereby participating in the symplasmic pathway during grain filling. The symplastic isolation of the nucellar epidermis and aleurone necessitates the presence of transporters, such as SWEET11/15, at the maternal/filial boundary. Meanwhile, ONAC127 is hypothesized to contribute to apoplasmic transport regulation by activating the expression of transporters like SWEET15 [40]. NF-YB1 is specifically localized in the aleurone layer, displaying stronger expression at the dorsal site, which is proposed to play a pivotal role in the apoplastic uptake of nutrients, including sugar into the endosperm, acting as a conduit bridging the maternal and filial tissues [41,42]. In this study, we found that grain-filling-associated genes, such as *GIF1* and *SWEET11/15*, were significantly downregulated in the caryopses of the *pex1* mutant compared to the WT (Figure 6C). These results suggest that the apoplastic pathway for assimilate translocation is altered in the *pex1* mutant, supporting the notion that OsPEX1 is involved in grain filling. In this study, we have not determined how OsPEX1 functions, but it may be through altered sugar transport.

Considering the subcellular localization (Figure 5) and tissue-specific expression pattern of OsPEX1 [10], along with the phenotype observed in the *pex1* mutant (Figure 1), it is proposed that OsPEX1 influences the transport of assimilates during grain filling, possibly through the symplastic pathway. However, its precise mechanism requires further elucidation.

## 4. Material and Methods

### 4.1. Plant Materials

Rice plants (*Oryza sativa* ssp. *Japonica*, cultivar Zhonghua 11, ZH11) were cultivated in a paddy field at South China Agricultural University (Guangzhou, China). The *pex1* mutant, as detailed in this paper, originated from an Activator/Dissociator (Ac/Ds) transposon-tagging population within ZH11 [43]. Spikelets were labeled on the branch to indicate flowering dates and were sampled daily during the 25-day period of caryopsis development.

### 4.2. Measurement of Grain Traits and Filling Parameters

Homozygous *pex1* plants were used for analysis of grain phenotypes. Grain thickness was measured with an electronic digital display Vernier caliper, and filled grains were used for measurement of 100-grain weight.

### 4.3. Histological Observation for Caryopsis

To examine the developing caryopsis, samples at 5 days after pollination (DAP), 7 DAP, and mature caryopses were immersed in FAA solution (50% ethanol: formaldehyde: glacial acetic acid = 89:5:6, with 6 mL glycerin per 100 mL FAA) overnight. Subsequently, they underwent dehydration in a series of ethanol gradients, followed by transferring into TO (TO-type biological transparent agent with turpentine as the main raw material (Beijing Solaibao Technology Co., Ltd., Beijing, China, catalog no. CB44716046) for transparency and ultimately embedding in paraffin.

To stain the starch, caryopses at 5 DAP and 7 DAP were treated with periodic acid–Schiff reagent [44]. In summary, the caryopses were transversely sectioned into 8-μm slices, dewaxed, and rehydrated. The tissue was subsequently oxidized in periodic acid solution for 15 min, rinsed in distilled water, exposed to Schiff’s reagent for 20 min, color-fixed using sulfite solution for 2 min, and washed with distilled water. Finally, the slides were dehydrated, mounted with neutral balsam, and then examined under a fluorescence microscope (Shunyu RX50, Yuyao, China). The analyses were performed with 4×, 10×, and 40× objectives; a DP27 digital camera; and bright-field/EPI-fluorescence (red channel with 560 nm excitation and 635 nm emission). Images were captured with a high-resolution CMOS camera.

To observe the caryopsis bran, the mature caryopses were subjected to the safranine O/fast green double-staining technique. The caryopses were sliced longitudinally into 8-μm sections; dewaxed; and gradually transferred into 100%, 95%, and 85% ethanol for 5 min each. Subsequently, they were stained with safranine O (dissolved in 70% ethanol) for 1.5 h and dehydrated with 70% and 85% ethanol for 1 min each. The caryopses were then coated with fast green (dissolved in 85% ethanol) for 40 s before undergoing a triple transfer into ethanol, gradually transitioning into TO. Finally, the specimens were mounted in balsam and visualized under an optical microscope (Olympus BX53, Tokyo, Japan). ImageJ software (version 1.8) was used to measure the thickness of bran at both dorsal and ventral sides of mature caryopses.

### 4.4. Subcellular Localization

To ascertain the cell wall localization of the OsPEX1 protein, the coding sequence of *PEX1* was amplified using primers PEX1-LRRF and PEX1-EXTR (Table S1). The length of the full genomic sequence of *PEX1* was 4200 bp (Figure S5). The resultant PCR product was then integrated into the *Hind*III/*Bam*HI sites of the pOX-eGFP vector and fused upstream of eGFP, generating the PEX1-eGFP fusion protein-expressing plasmid pOX-PEX1-eGFP. The promoter of the pOX vector is ubiquitin, while its terminator is Nos 3′. Subsequently, this construct was introduced into *Agrobacterium tumefaciens* EHA105 via electroporation and infiltrated into young leaves of *Nicotiana benthaniana* at the four-leaf stage. Centrifuge the *Agrobacterium* cells and resuspend them in the infiltration buffer (10 mM MgCl_2_, 10 mM MES, pH 5.6, and 100 μM Acetosyringone) to an OD_600_ of approximately 0.5–1.0. Use an unspiked syringe to infiltrate the bacterial solution from the back side (lower epidermis) of the tobacco leaf, avoiding damage to the leaf. After an incubation period of 48–60 h, visualization was conducted using a confocal microscope (Leica TCS SP8, eGFP with an excitation peak at 489 nm and an emission peak at 508 nm; mCherry with an excitation peak at 587 nm and an emission peak at 610 nm).

For determining whether the PEX1 localizes to the endoplasmic reticulum (ER), the N-terminal LRR domain of OsPEX1 was amplified utilizing the primer pair PEX1-LRRF and PEX1-LRRR (Table S1). This region was cloned into the *Nhe*I/*Bam*HI site of the pAN580 vector, resulting in the creation of pAN580-LRR-eGFP. The pAN580 vector utilizes the 35S promoter and also employs Nos 3′ as its terminator. Subsequently, this construct was introduced into rice protoplasts along with ER (CD3-960-mCherry) or plasma membrane (CD3-1008-mCherry) markers [45] for co-localization analyses.

Protoplast preparation and transformation were conducted as follows: rice seeds were germinated for 7–14 days until the resulting plants reached an approximate height of 4–8 inches. The seedlings were finely sliced into 0.5 mm pieces, and the tissue was digested with cellulase RS and macerozyme in the dark while being gently shaken. The protoplasts were then filtered through a 35 µm nylon mesh. An equal volume of W5 solution was added, and the mixture was centrifuged at low speed to isolate the protoplasts. The cells were counted using a hemocytometer and resuspended in MMG solution. For transformation, 3 µg of plasmid DNA was used to transfect 2 × 10^5^ protoplasts with PEG4000 for 15–20 min. The cells were subsequently rinsed, resuspended in W5 solution, and incubated in the dark overnight.

### 4.5. qPCR and RNA-Seq

Total RNA was extracted from 7 DAP caryopses using the MagZol reagent, following the manufacturer’s instructions (Magen, Guangzhou, China). The extracted RNA was then used to synthesize cDNA with TransScript^®®^ One-Step gDNA Removal and cDNA Synthesis SuperMix (TransGen, Beijing, China). Quantitative PCR (qPCR) was performed using TransStart^®®^ Green qPCR SuperMix (TransGene Biotech, Beijing, China) according to the manufacturer’s protocols. Gene expression levels were determined using the 2^−∆∆Ct^ relative quantification method by employing the Ct values obtained by RT-qPCR [46], with 25S rRNA as the endogenous control gene. The primers used for qPCR are listed in Table S1.

For RNA-seq analysis, total RNA was extracted from three biological replicates of 7 DAP wild type (WT) and *pex1* mutant caryopses using MagZol reagent (Magen, Guangzhou, China). Subsequently, RNA-seq libraries were prepared with TruSeq RNA Library Preparation Kit (Illumina, San Diego, CA, USA) following the manufacturer’s recommendations, and index codes were incorporated to attribute sequences to each sample. The resulting libraries underwent sequencing on an Illumina Novaseq platform to generate 150 bp paired-end reads. The obtained reads were aligned to the *Oryza sativa* spp. *japonica* cv. Nipponbare reference genome assembly (IRGSP-1.0) utilizing hisat2 software [47], and gene annotation was performed based on rice databases (MSU7, RAP-DB) [48,49]. FPKM values were computed to identify differentially expressed genes (DEGs) (*padj* < 0.05, |log_2_fold-change| > 1.0) using DEseq2 software (version 1.38.3) [50]. Additionally, GO enrichment analysis of the DEGs was conducted using the clusterProfiler enrichment tool [51], and the DEGs were visualized with TBtools-II (version 2.096) [52].

### 4.6. Gene Accession Number

LOC_Os11g43640 (*PEX1*), LOC_Os09g12660 (*AGPS1*), LOC_Os08g25734 (*AGPS2*), LOC_Os01g44220 (*AGPL2*), LOC_Os07g22930 (*GBSSII*), LOC_Os06g06560 (*SSI*), LOC_Os06g12450 (*SSIIa*), LOC_Os02g32660 (*BEIIb*), LOC_Os08g40930 (*ISA1*), LOC_Os03g55090 (*Pho1*), LOC_Os04g33740 (*GIF1*), LOC_Os02g30910 (*SWEET15*), LOC_Os05g33570 (*FLO4*), LOC_Os03g18310 (*FLO23*), LOC_Os06g13810 (*PFP1β*), LOC_Os10g33800 (*FLO16*; *MDH10.1*), LOC_Os12g14070 (*FLO11*; *HSP70-2*), LOC_Os10g25130 (*FLO12*; *AlaAT1*), LOC_Os02g57180 (*FLO13*; *NDUFA9*), LOC_Os05g07720 (*TAR1*), LOC_Os01g16714 (*YUC9*), LOC_Os02g49410 (*NF-YB1*), LOC_Os05g39990 (*EXP4*), LOC_Os10g40720 (*EXPB3*), LOC_Os04g49410 (EXPA10), and LOC_Os11g31340 (*ONAC127*).

## 5. Conclusions

The *pex1* mutant exhibited abnormal starch accumulation in the pericarp during the filling phase, along with atypical nucellar projection cells. These observations suggest a defect in sugar transport from the dorsal vascular bundle to the aleurone cells. This evidence indicates that the LRX protein OsPEX1 plays a crucial role in grain filling.

## Data Availability

The data presented in the study are deposited in the NCBI repository, accession numbers of WT (ZH11, SAMN41710461) and *pex1* mutant (*pex1/pex1*, SAMN41710462) available at https://www.ncbi.nlm.nih.gov/biosample/ (accessed on 6 June 2024).

## Supplementary Materials

The following supporting information can be downloaded at https://www.mdpi.com/article/10.3390/plants14172723/s1: Figure S1: GUS staining on the OsPEX1pro::GUS lines at 7 days after pollination; Figure S2: Subcellular localization of OsPEX1 in rice protoplasts; Figure S3: Expression of cell wall extensibility associated genes quantified by RNA-seq analysis; Figure S4: Schematic diagram of the simple type in plasmodesma; Figure S5: The full genomic sequence of *OsPEX1* gene; Table S1: Primers used in this study; Table S2: Differentially expressed genes associated with grain filling at 7 DAP.
