# Supplementary material for: Extensin-like Protein OsPEX1 Modulates Grain Filling in Rice
Source: Plants (Basel). 2025 Sep 1;14(17):2723. doi: 10.3390/plants14172723 (PMC12430520; doi:10.3390/plants14172723)
Supplement: Supplementary file 1 [file plants-14-02723-s001.zip › Figure S1-S5.pdf]

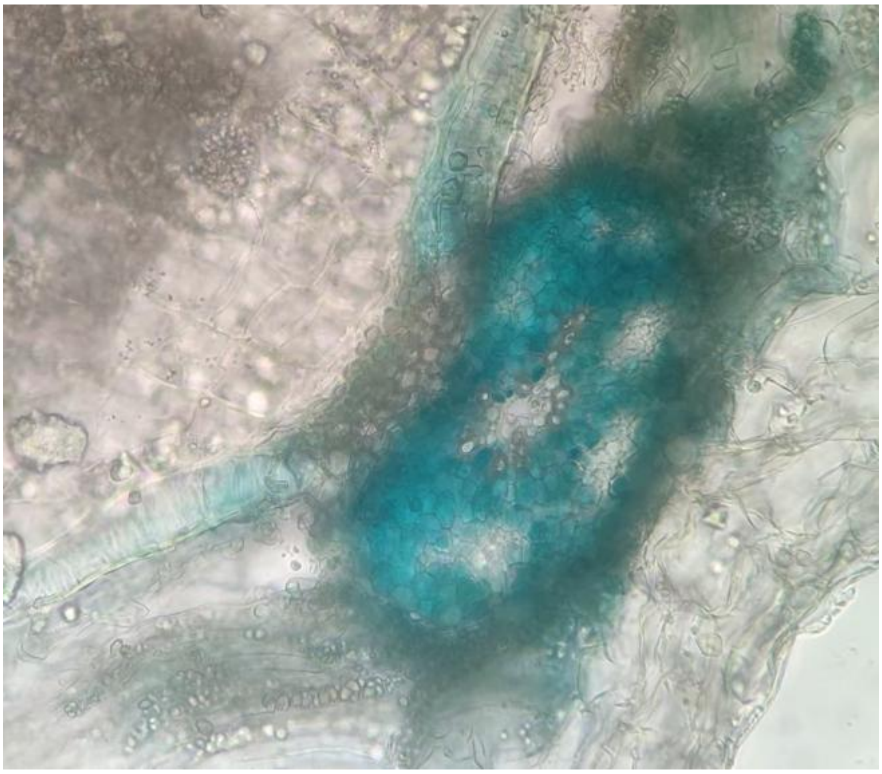

Figure S1. GUS staining on the OsPEX1pro::GUS lines at 7 days after pollination.

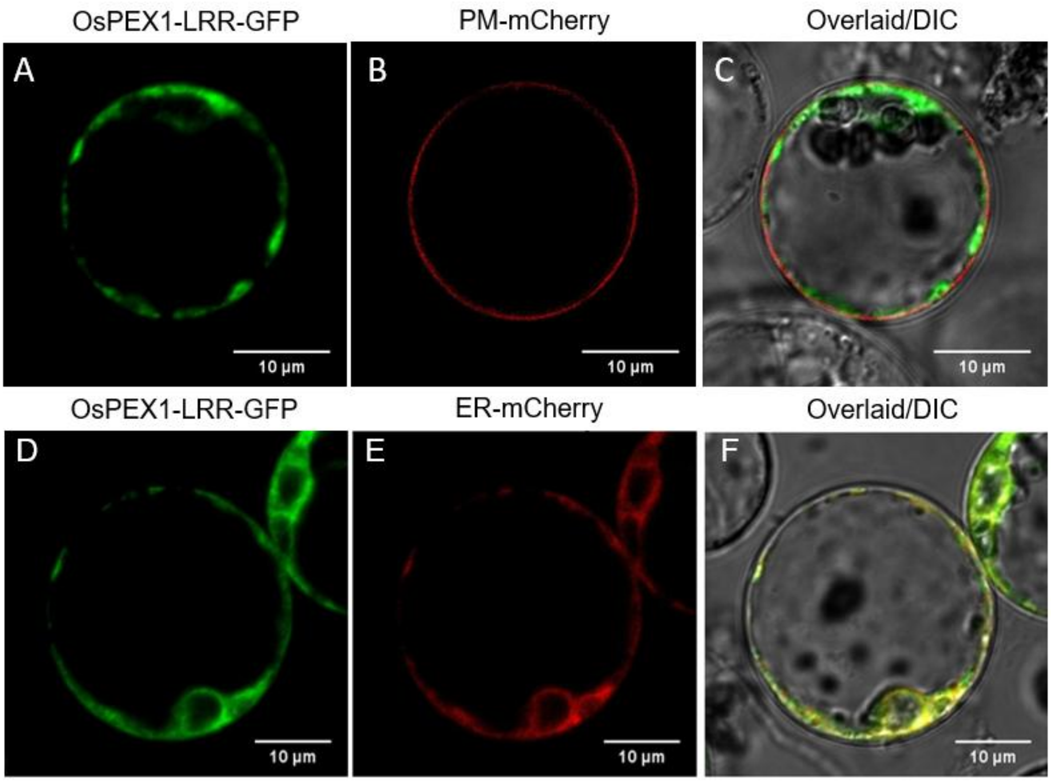

Figure S2. Subcellular localization of OsPEX1 in rice protoplasts. (**A-C**) the localization of N-terminal half of OsPEX1 with plasma membrane marker. (**D-F**) the localization of N-terminal half of OsPEX1 with endoplasmic reticulum marker. GFP fusions to OsPEX1 proteins are shown in green; Plasma membrane marker (PM-mCherry) was shown in red.

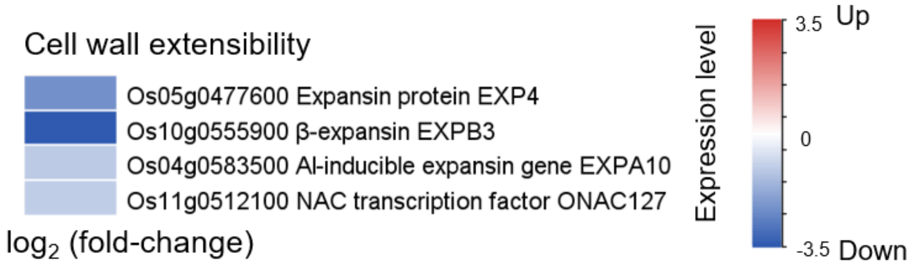

Figure S3. Expression of cell wall extensibility associated genes quantified by RNA-seq analysis. The $\log_2$ fold-change values between *pex1* mutant and the WT were calculated from RNA-seq data and are shown as a heat map.

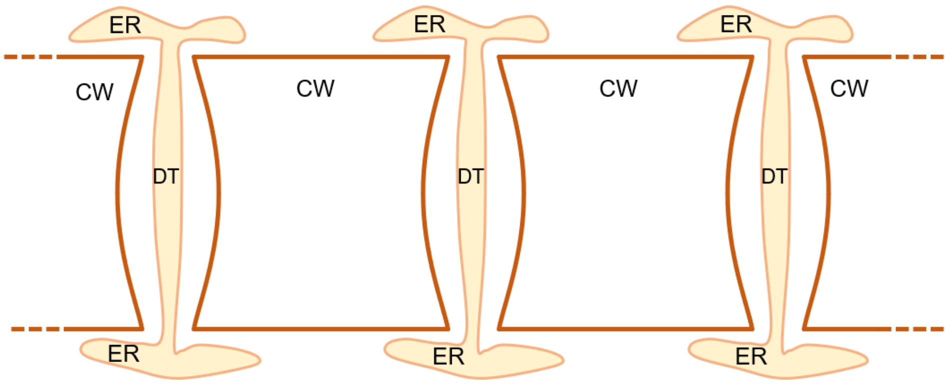

Figure S4. Schematic diagram of the simple type in plasmodesma. CW, cell wall. DT, desmotubule. ER, endoplasmic reticulum.

> *OsPEX1*

ATGGACCTCCGGCTCCTCCTCGCCCCCGGCGGGCGCCGCGCCGCCATGGCACTGATTCTCGCCGTCCTCGCCGCCT
GCCTCTCCTCCGCCGCCAACGTCGGCGCGGTGACCAGCGCCGAGGTGTCGTACATCGCGCACCGGCAGCTGCTGGC
GATGAAGGAGGCCGGCGTGAGCGAGGAGGGCGACCTGCCGTCCGATGACTTCGACTTCGACGACCGCGTCGGAGTC
GCCGTCGGCGACTTCCCGAACCCGCGGCTCCGCAAGGCGTACATCGCGCTCCAGGCGTGGCGGCGCGCGTTCTACT
CGGACCCCAAGGGGTACACCAACAACTGGACGGGCAACGACGTGTGCTCCTACAACGGCGTCATCTGCTACGCCGC
CATCGACGACCCCAAGATCATGGTGGTCGCCGGCATCGACCTCAACGGCGCCGACATCGCCGGGTACCTCCCGCCG
GAGCTCGGCCTCCTCACCGACCTCGCCTTCTTCCACATCAACACCAACCGCTTCTGCGGCATCATCCCCAAGAGCA
TGTCGAGGCTGTCGCTGCTGCACGAGTTCGACGTCAGCCACAACCGCTTCGTCGGCGTCTTCCCCCACGTCTGCCT
CGAGATGGCCGTGCTCAAGTACCTCGACATCCGCTTCAACGACTTCGAGGGCGAGCTGCCGCCGGCGCTGTTCGAC
AAGGAGCTCGACGCCATCTTCGTCAACAGCAACCGATTCGTCGGCTACATCCCGGGCAACCTCGGCAACTCCACCG
CCTCCGTCATCGTCTTCGCCAACAACGCCTTCGTCGGCTGCATCCCCAAGAGCATCGGCTGCATGGCCAAAACGCT
CGACGAGATCAGCTTCATGAACAACAAGCTCGACGGCTGCGTGCCCATGGAGATGGGCTACCTGCAGAACACCTAC
GTCATCGACATCAGCGGCAACGTCCTCGTCGGCACGCTGCCCACCTCGCTCTCCAACTGCAGCAAGCTGGAGCAGC
TGGACGTGTCCAGGAACGTCTTCACCGGCATCGTCCACGAGTCCATCTGCGAGCTCCCCGTGCTCGTCAACTTCAG
CTTCGCCTACAACTTCTTCAACTCCGAGTCGGCGCCGTGCATGCCGTCGGAGAGCAGCAAGGTCAACCTCGACGAC
AAGGACAACTGCCTCGGCGCGCTCCGACCGGCGCAGAAGACGACGCTGCAGTGCGCCCCCGTGCTCGCCCGCCCCG
TCGACTGCAGCAAGCACGTCTGCCCCGGACACCCGACGCCCGGGAAGCCGTCGGAGCCGCCGGAGAAGCCGCCGCT
CATCCCGGTGCCGGTGGGGCCGCCGGAGAAATCGCCGGCTTACGAGGAACCACCGGCCGCGCCTTCCACCCCGACG
TCGCACGGCCCGCCACCTCCAGAGGAGGAGTCACCTGAGGAACCACCCGAGGAACCAACACCGTCGCCGACACCAA
GCAGCCCAGAGTCACCGGCCAAGATGGCGCCGCCTCCGGCACCCGCCATCAAGGGAGTGACATCGCCGCCGGCAGA
GTATGGTGCTCCACCGCCACCAAGTTCGGGTTGGCTCCCCAAGAGTCCCGAGCGCAAGAAGGCACCTCCGCCACAA
GCGGAGCCTCCTACTGAGTACTCCCCACCTGCAACTCCAGAGAGCTCGCCACCACCTGAAGGGAAGTCCCCTCCTA
CGCCGACGGCCTCGCACTCGCCACCACCGGTACCAGAGGGCCACACACCTTCCCCACCAAAGTCGGACCACCTGC
TGGGGAGTCTCCTCCTACACCTGAGTCAAAAGCCTCGCCTCCACCGACTCCAGAAGAATACACACCTTCCCCGCCA
AAATCGACACCACCAGCTGAGAAGTCTCCTCCTACACCTGAGTCGAAAGCCTCATCTCCACCACCACCCGCTCCAG
AGGGCCACACACCTTCCCCGCCAGAGTCGACACCACCATCTGAGAAATCTCCTCCTACACCCGAGTCGAAAGCCTC
ATCTCCACCACCACCCACTCCAGAGGGCCACACACCTTCCCCGCCAAAGTCGACACCGCCAACTGAGAAGTCTCCT
CCTACACCTGAGTCGGAATCCTCCTCTCCACCACCACCCGCTCCAGAGGGCCACATGCCTTCCCCGCCAAAGTCGA
CGCCACCAGTTGAGAAGTCTCCTCCCACGCCTGAGTCGGAAGCCTCATCTCCACCACCACCCGCTCCGGAGGGCCA
CACACCTTCCCCACCAAAGTCATCACCACCAGAAGAGAAGTCTCCTCCTATACCGCCGACCTCGCATACATCACCT
CCAACTCCAGAGGAATACACACCTTCCCCACCAAAGTCATCGCCACCAGAAGAGAAGTCTCCTCCACCACATTCCC
CAGAAAAGTCACCACCATCAGAGGCTCACCCAACTTCTCCACCTCCCTCGGAGAAGTCACCTCCAACACCAGCTGA
AGAGAGTTCTCCGCCAACTCCGGAAAAATCTCCATCACCACCATCGGGTCATGAAGGCACTCCACCATCCCCAGTG
AAATCTTCCTCACCACCACCAGAAGCTCATGTTAGCTCACCACCACCAGAAAAATCTTCCTCGCCACCACCAGAAG
CTCATGTTAGTTCACCACCACCACCTGAAAAGTCTCCACCACCACCAGAGACAAAGTCTCCACCAACGCTAACACC
GGAGATCTCTCCACCTCCGGAAGGGAAGTCCCCACCATCACATACTCCGGAGAGCTCATCCCCACCATCTAAGGAG
TCAGAACCACCACCGACACCAACACCAAAGAGCTCTCCACCATCTCACGAGGAGTACGTTCCTCCATCTCCGGCAA
AATCAACTCCACCTCCAGAAGAGAAGTCCCCACCATCACATACTCCGGAGAGCTCATCCCCACCATCTGAGGAGTC
AGAACCACCACCGTCACCAACACCAAAGAGCTCTCCACCATCTCACGAGGAGTACGTTCCTCCATCTCCGGCGAAA
TCAACTCCACCTCCAGAAAAGCCACTACCACCACACACACCAACAATAAATAGTTCTCCACCATCGGAAGAAGAGT
ACATGCCTCCATCTCCAGTGAAATCAAGTCCACCACCAGCCGAGAAGTCCCAGCCACCGCCATCTCCAGTTGAGTC
AGTACTTCCACCAGTGAAGTCTTCACCACCACACGCACCGGTTATCTCAGAACCACCACCAACAAAGTCTTCACCG
CCGCAAGTGCCAGTGACCTCGGAACCACCGCCAGCAAAGTCTTCACCTCCACATGAACCAATTAGCCCGCCAGAAA
CACCAGAGAAGTCTTACCCGCCATCAACTCCTGAGGAATCATCTCCTCCCTCAGTCCCCAAGGCTTCATCTCCACC
AACTGAGAAGTCTCTTCCTCCACCGGCTACAGTGAGCTTGCCGCCTCCAACAGTTAAGCCTTTGCCCCCACCGGTT
CCTGTGAGCTCACCGCCACCTCCGGAGAAGTCTCCACCCCCGCCAGCTCCGGTGATCTTGCCGCCTCCTCCAATTA
AATCTCCTCCCCCACCGGCTCCAGTCATCTCGCCACCTCCTCCAGTTAAGTCCCCACCACCACCAGCACCAGTTAT
CTTGCCACCTCCTCCTGTGAAGTCTCCACCACCACCAGCACCGGTCATCTCGCCACCCCCACCTGTGAAGTCTCCA
CCTCCTCCCGCACCAGTCATCTTGCCACCCCCACCTGTGAAGTCTCCACCTCCACCTGCACCGGTCATCTCGCCAC
CACCTCCAGAGAAGTCACCACCACCAGCAGCACCTGTGATCTTGTCACCACCAGCGGTGAAGTCTCTTCCCCCACC
GGCACCGGTCAGCCTACCACCACCACCGGTGAAGTCGCTTCCCCCACCAGCACCGGTCAGCCTACCACCACCCGTC
GTGAAGTCACTTCCCCCACCAGCACCAGTCAGCCTACCACCACCAGCGGTGAAGCCTCTTCCCCCACCAGCACCAG
TCAGCCTACCACCACCAGCGGTGAAGCCTCTTCCCCCACCAGTTCCGCAGGTCTCCCTCCCCCCACCGAAACAGGA
GTCATTGCCGCCACCAGCAAAGGAAGCTGAAGCTCCACCTGCAAAGGAATCTAAACCTCCACCAGCAATGGAAGCT
GAAGCTCCACCGGCGTTCGACACTACTGTACTCTTACCGCCGGTGATGGCGCACCAGTACGCCTCACCTCCACCAC
CTCAGTTCCAAGGATACTAA

Figure S5. The full genomic sequence of *OsPEX1* gene.
