# Supplementary material for: Extensin-like Protein OsPEX1 Modulates Grain Filling in Rice
Source: Plants (Basel). 2025 Sep 1;14(17):2723. doi: 10.3390/plants14172723 (PMC12430520; doi:10.3390/plants14172723)
Supplement: Supplementary file 1 [file plants-14-02723-s001.zip › Table S1-S2.pdf]

Table S1. Primers used in this study

Gene	MSU_Locus	Primers	Sequence (5′→ 3′)
PEX1	LOC_Os11g43640	PEX1-LRR-F	GCTAGCAAGCTTATGGACCTCCGGCTCCTCCT
		PEX1-LRR-R	GGATCCTCTAGAGCAGACGTGCTTGCTGCAGT
PEX1	LOC_Os11g43640	PEX1-EXT-F	ACTAGTCCCGGACACCCGACGCCCGGGAA
		PEX1-EXT-R	GGATCCGTATCCTTGGAACTGAGGTG
GIF1	LOC_Os04g33740	GIF1-F	GATCATGTACACCGGCGTCA
		GIF1-R	CAGGTCGAGGCTGTTCTTGA
SWEET15	LOC_Os02g30910	SWEET15-F	AAGAAGTCGACGGAGGGGTT
		SWEET15-R	CGAAGAGGCCGATGTTGAGG
LTPL36	LOC_Os03g25350	LTPL36-F	GTTTGTTCTTGGTCCTCGCC
		LTPL36-R	CTTCTCCATGCACCACACCT
NF-YB1	LOC_Os02g49410	NF-YB1-F	AAGAAGGCGGCAGTGAGATC
		NF-YB1-R	GCCGAATGAGCCCAAGTAGT
ONAC127	LOC_Os11g31340	ONAC127-F	GAACGAACGCCTTGGCAAC
		ONAC127-R	CTCGTCACAGGGCAAGGTAT
AGPL2	LOC_Os01g44220	AGPL2-F	GCACAACCAATGAGAAGGGC
		AGPL2-R	AGGAGTGTCTCTATCAGCATCTG
25S rRNA		25SR-F	AAGGCCGAAGAGGAGAAAGGT
		25SR-R1	TTGGCGGGCCGTTAAGCAGAAAAGA

Table S2. Differentially expressed genes associated with grain filling at 7 DAP

Gene_id	WT_readcount	pex1_readcount	log2FoldChange	pval	padj	Significant
Sugar transport/synthesis						
Os01g0919400	1995.313562	1625.939565	-0.29284	0.41897	0.59892	FALSE
Os03g0218400	30.85977383	191.306211	2.6356	0.000020997	0.00012295	TRUE
Os07g0559700	3099.011499	2047.681123	-0.59668	0.07821	0.16608	FALSE
Os05g0187100	1172.820955	486.3079937	-1.2616	0.00024701	0.0011327	TRUE
Os03g0170900	11450.23593	10469.69455	-0.12882	0.57559	0.73353	FALSE
Os12g0641400	1766.370094	1765.686566	0.0021592	0.9891	0.99525	FALSE
Os02g0576600	36.26187149	59.23042932	0.67883	0.074582	0.15971	FALSE
Starch synthesis and translocation						
Os09g0298200	26832.52717	9205.877553	-1.5432	7.0872E-30	9.3205E-28	TRUE
Os08g0345800	95477.43605	19554.3474	-2.2879	6.4514E-16	1.9706E-14	TRUE
Os01g0633100	108664.5377	22063.28372	-2.3002	9.9361E-15	2.5807E-13	TRUE
Os07g0412100	2145.934317	574.5762073	-1.9074	7.3267E-48	3.794E-45	TRUE
Os06g0160700	24551.50472	8648.038203	-1.5052	5.6527E-14	1.3381E-12	TRUE
Os06g0229800	35548.1602	5806.247681	-2.6135	1.7166E-63	2.8445E-60	TRUE
Os02g0528200	241123.0649	49375.93756	-2.288	4.8584E-48	2.597E-45	TRUE
Os08g0520900	79106.60495	26282.81039	-1.5897	7.0996E-43	2.7681E-40	TRUE
Os03g0758100	130902.2327	19958.78283	-2.7135	1.4665E-150	4.8603E-146	TRUE
Os04g0413500	4104.835764	667.860689	-2.6154	2.0737E-08	2.1537E-07	TRUE
Os02g0513100	1657.259717	637.324566	-1.3809	8.1239E-13	1.6397E-11	TRUE
Os03g0735000	8878.225306	4658.208314	-0.93194	1.3001E-07	1.1709E-06	FALSE
Os06g0133000	139526.6338	79553.94717	-0.81062	0.00015007	0.00072478	FALSE
Os02g0744700	293.1368252	188.1271144	-0.6219	0.11533	0.22605	FALSE
Os08g0191433	30380.31221	25177.69412	-0.27113	0.22591	0.38342	FALSE
Os04g0624600	1787.560096	1648.10472	-0.1158	0.43824	0.61753	FALSE
Os11g0508600	1329.295353	1536.572292	0.20662	0.64264	0.78281	FALSE
Os02g0301100	2571.963931	6682.953498	1.3771	1.7793E-12	3.4559E-11	TRUE
Os09g0508250	848.942728	2308.57184	1.4428	5.7366E-07	4.6134E-06	TRUE
Os01g0606000	1911.433448	1018.165104	-0.90513	0.011016	0.032125	FALSE
Os08g0535200	41449.68137	40195.39285	-0.044276	0.83754	0.91037	FALSE
Floury endosperm associated						
Os05g0405000	549420.4985	116556.5754	-2.2368	2.5593E-39	7.6413E-37	TRUE
Os03g0294200	2150.633381	1054.05468	-1.0288	1.3535E-10	2.0043E-09	TRUE
Os06g0247500	11631.69067	3089.398767	-1.9111	1.773E-15	5.0743E-14	TRUE
Os10g0478200	46261.15557	7689.98115	-2.5881	1.4303E-34	2.9442E-32	TRUE
Os12g0244100	51461.40831	21958.92177	-1.2289	3.1307E-07	2.6313E-06	TRUE
Os10g0390500	95308.6292	24568.10652	-1.9557	6.8507E-15	1.8178E-13	TRUE
Os02g0816800	3035.359411	1504.382903	-1.0103	3.3844E-10	4.7127E-09	TRUE
Os04g0645100	10339.44524	9890.573034	-0.064083	0.82114	0.90528	FALSE

(Continued to Table S2)

Gene_id	WT_readcount	pex1_readcount	log2FoldChange	pval	padj	Significant
Lipid metabolism and transport						
Os03g0232800	1785.309853	236.6542589	-2.9007	4.5651E-12	8.3772E-11	TRUE
Os12g0114500	807.965889	327.2103622	-1.3102	6.4679E-10	8.6225E-09	TRUE
Os12g0114800	12019.70834	2489.652181	-2.2696	9.8388E-13	1.9631E-11	TRUE
Os03g0369100	23929.95726	2624.419412	-3.1882	0.000026641	0.00015233	TRUE
Auxin biosynthesis						
Os05g0169300	2179.192857	900.5060658	-1.2703	2.9101E-08	2.9511E-07	TRUE
Os01g0273800	4642.118357	2303.926262	-1.0099	9.6732E-09	1.069E-07	TRUE
Os02g0725900	14242.98124	4119.815922	-1.7889	1.3884E-09	1.7642E-08	TRUE
Os12g0189500	469.3495961	585.7888566	0.31117	0.27413	0.4443	FALSE
Protein biosynthesis						
Os02g0242600	231164.1332	35169.05998	-2.7164	3.3706E-23	2.2985E-21	TRUE
Os10g0400200	1317438.162	3583884.562	1.4438	4.3183E-07	3.5359E-06	TRUE
Os02g0268300	566710.3686	1508154.075	1.4121	3.4388E-06	0.000023822	TRUE
Os08g0127900	34450.38897	137179.6267	1.9935	7.5246E-06	0.000048516	TRUE
Os02g0115900	42646.71256	16590.21402	-1.362	0.0013059	0.0049791	TRUE
Os11g0199200	108153.5632	24288.30312	-2.1546	3.1776E-25	2.646E-23	TRUE
Os09g0451500	6686.39119	1852.1626	-1.8508	6.8049E-31	9.8912E-29	TRUE
Os03g0427300	1119128.782	1735885.164	0.63329	0.0044489	0.014648	FALSE
Os02g0249800	691144.0171	645385.4587	-0.098825	0.60855	0.76163	FALSE
Os02g0249000	68803.80254	123664.5714	0.84586	0.0023711	0.0084541	FALSE
Os02g0453600	485854.5312	714562.8805	0.55653	0.02511	0.064707	FALSE
Os05g0367800	1.404811307	0	-2.4365	0.41436	0.59496	FALSE
Amino acid metabolism						
Os03g0712800	3927.401865	22885.16628	2.5426	1.3616E-16	4.5765E-15	TRUE
Cell wall extensibility						
Os05g0477600	4724.645247	947.2396511	-2.3127	9.7143E-18	3.8055E-16	TRUE
Os10g0555900	17735.50808	1624.368872	-3.4478	1.9115E-06	0.000013898	TRUE
Os04g0583500	1694.882009	791.9221339	-1.0974	6.2625E-12	1.1268E-10	TRUE
Os11g0512100	821.6481808	402.8394484	-1.0373	1.2919E-06	9.6847E-06	TRUE
Os03g0132200	940.443896	2163.191403	1.2028	3.0499E-08	3.0835E-07	TRUE
Os11g0512600	4620.828497	3629.720321	-0.34905	0.17608	0.31603	FALSE
